# Why women choose to deliver at home in India: a study of prevalence, factors, and socio-economic inequality

**DOI:** 10.1186/s12889-021-11779-5

**Published:** 2021-10-02

**Authors:** Ratna Patel, Strong P. Marbaniang, Shobhit Srivastava, Pradeep Kumar, Shekhar Chauhan

**Affiliations:** 1 Department of Public Health and Mortality Studies, International Institute for Population Sciences, Mumbai, India; 2 Department of Mathematical Demography and Statistics, International Institute for Population Sciences, Mumbai, India; 3 Department of Population Policies and Programmes, International Institute for Population Sciences, Mumbai, India

**Keywords:** Place of delivery, Home delivery, Socio-economic inequality, India

## Abstract

**Background:**

To promote institutional delivery, the Government of India, through the Janani Suraksha Yojana (JSY) program, gives monetary reward to all pregnant women who give birth at the government or private health center. Despite providing cash assistance, a higher number of women are still preferring delivering at home. Therefore, this study sought to determine the prevalence of home births and identifying the factors influencing women’s choice of home deliveries.

**Methods:**

Data from the National Family Health Survey (NFHS) conducted during 2005–06 and 2015–16 were used in the study. The respondents were women 15–49 years; a sample of 36,850 and 190,898 women in 2005–06 and 2015–16 respectively were included in the study. Multivariate logistic regression was used to determine the factors influencing home delivery. Income-related inequality in home delivery was quantified by the concentration index (CI) and the concentration curve (CC), and decomposition analysis was used to examine the inequality in the prevalence of home deliveries.

**Results:**

The prevalence of home deliveries has reduced from 58.5% in 2005–06 to 18.9% in 2015–16. The odds of delivering babies at home were lower among women who had full ANC in 2005–06 [AOR: 0.34; CI: 0.28–0.41] and in 2015–16 [AOR: 0.41; CI: 0.38–0.45] and were higher among women with four or higher parity in 2005–06 [AOR: 1.70; CI: 1.49–1.92] and in 2015–19 [AOR: 2.16; CI: 2.03–2.30]. Furthermore, the odds of delivering babies at home were higher among rural women and were lower among women with higher education. It was found that the value of CI increased from − 0.25 to − 0.39 from 2005-06 to 2015–16; this depicts that women delivering babies at home got more concentrated among women from lower socio-economic status.

**Conclusion:**

There is a need to promote institutional deliveries, particular focus to be given to poor women, women with higher parity, uneducated women, and rural women. ANC is the most concurring contact point for mothers to get relevant information about the risks and complications they may encounter during delivery. Therefore, effort should be directed to provide full ANC. Targeted interventions are called for to bring improvements in rural areas.

## Background

The choice of place of delivery has been mostly found to be associated with maternal and neonatal outcomes. Maternal and neonatal mortality from inadequate health services has been identified as the global challenge that has seen Southern Asian countries contribute about 20% of global maternal deaths and 38% of global neonatal deaths in 2017 [1, 2]. Most authors highlighted that the factors associated with these maternal and fetal deaths are the occurrence of home deliveries as they are mostly unplanned, accidental, and unhygienic [3, 4]. According to the latest estimates, over 800 women worldwide died every day from complications in pregnancy and childbirth [1]. These complications usually arise during delivery and are difficult to predict but can be effectively managed, and deaths can be prevented through delivery at the health facility equipped with skilled birth attendants placed in an enabling environment [5]. Moindi et al., (2015) acknowledge that skilled birth attendant during childbirth in a hygienic environment with necessary skills and equipment to identify and manage any emerging complications reduces the likelihood of women and child died during the delivery process [6]. Most pregnancy and birth complications are timely manage in the health facility [7], unlike home delivery where women are not attended by the skilled birth attendant, and the chances of complications resulting in death are high [8, 9].

To promote institutional delivery, the Government of India, through the Janani Suraksha Yojana (JSY) program, provides a certain amount of money to all pregnant women who give birth at the government or private health centre [10]. An evaluation of this JSY program in 2007–2008 shows an increase in Antenatal Care (ANC) visits and institutional delivery [11]. However, this has not translated into a reduction of maternal and neonatal mortality rates [12], as these rates are still being reported significantly high in India [1, 2]. The global estimate shows that there were 295,000 maternal deaths in 2017; India alone contributes about 12% (35,000) of global maternal deaths [1] and about 26% of the global neonatal deaths [13].

Many authors in India have argued the existing disparity in terms of utilization and accessibility to maternal healthcare services among the socially marginalized group [14], across states [15], and among the poor and the non-poor [16]. Studies related to child home delivery have argued that factors influence the choice of place of delivery [17–19]. The significant factors that have been identified are distance to health facilities [6], hospitalization bills and transportation cost [20, 21], level of knowledge, and access to antenatal care [22, 23]. Das & Hammer (2014) explained that people were not using institutional delivery because of the low quality of health facilities [24]. Education is an important factor influencing the choice of place of delivery [16]. Educated couples may be more open to modern medicines, aware of the importance of skilled birth attendants, and more comfortable communicating with the health attendant [25]. A study in India found that economic factors such as spousal occupation and monthly income influence the decision on delivery [18]. For example, Sarkar et al. (2018) mentioned that women prefer home delivery because the amount receives from the government incentive (JSY) is less than the transportation, flooding, and lodging expenses of attendants [26]. Further, among the rural women, the fear and embarrassment of giving birth in the presence of a stranger at the hospital, most women decided to give birth at home as they received better care at home [18, 27, 28]. The abuse experience dduring child delivery, such as physical and mental abuse, verbal abuse, denial of hospital admission, and untimely delay of treatment in the government or private hospitals, could promote home deliveries, especially among the lower socio-economic groups [28, 29].

Recent literature from India [14–16, 30] have highlighted the important factors which act as the main barrier for accessing maternal healthcare services. However, there is a dearth in the study that assesses the prevalence and determinants of child home delivery in India using a large-scale survey. Even though evidence from India reported the regional and state level inequality in the use of maternal health services [31], little knowledge is known about the socio-economic inequality in women delivery babies at home. Economic status is the major contributor to inequality in achieving acceptable levels of institutional delivery in India [15, 27]. Despite government of India initiative through cash incentives to promote institutional delivery, still many women deliver at home and many think that institutional delivery is not necessary [32]. With this background, the current study aimed to determine the prevalence of childbirth at home and its associated socio-economic risk factors. Further, the study will assess the socio-economic status inequality for women delivering babies at home. Our findings will be important for the public health researchers and policy maker to develop effective intervention measures that targets vulnerable sections of women and improve access to institutional delivery and maternal health services.

## Methods

### Data

The data for this study came from the NFHS-3 and NFHS-4 rounds of the National Family Health Survey (NFHS), which were conducted in 2005–06 and 2015–16, respectively. The Nationwide Family Health Survey (NFHS) is a cross-sectional national representative survey undertaken by the Ministry of Health and Family Welfare (MoHFW) of the Government of India. It contains statistics on India’s population, health, and nutrition for each state and union territory. In NFHS-3, 124,385 women aged 15 to 49 years were interviewed, but in NFHS-4, 601,509 households and 699,686 women aged 15 to 49 years were interviewed. To choose the sample, the survey utilised a two-stage stratified sampling technique, with the sampling frame derived from the national census to pick main sampling units (PSUs). In rural regions, PSUs were villages, and in urban areas, Census Enumeration Blocks (CEBs). PSUs with fewer than 40 homes were connected to the PSU closest to them. Within each rural stratum, villages were chosen from the sample frame with a probability proportionate to size (PPS). The survey reports covered the methodology, sample strategy, and data collecting technique [33, 34]. The effective sample size for the study was 36,850 and 190,898 women aged 15–49 years who gave last birth during 5 years preceding the survey for NFHS 2005–06 and 2015–16, respectively.

### Outcome variable

The question was asked to women, ‘Where did you give birth to (NAME)?’ The responses were home (included your home, parents’ home, and other home), the public health sector (included govt./municipality hospital, uhc/uhp/ufwc, government dispensary, chc/rural hospital/block phc, phc/additional phc, sub-center, other public sector health facility) and private (included hospital/maternity home/clinic, other private sector health facility, NGO or trust hospital/clinic, other). The outcome variable was dichotomous and coded as ‘1’ if women delivered at home and ‘0’ otherwise.

### Predictor variables

The predictors included age at first birth (< 18 years, 18–24 years and 25 years or more), parity (first, second, third, and four or more), antenatal care (no, partial and full), mass media exposure (no and yes), educational attainment (no schooling, primary, secondary, and higher), caste (Scheduled Caste, Scheduled Tribe, Other Backward Class, others), religion (Hindu, Muslim, and others), wealth index (poor, middle, rich), place of residence (urban and rural), and region (North, Central, East, Northeast, West, and South). Full ANC is defined as four or more antenatal visits. Women’s exposure to mass media (how often they read newspapers, listened to the radio, and watched television; responses on the frequencies were: almost every day, at least once a week, less than once a week, or not at all; women were considered to have any exposure to mass media if they had exposure to any of these sources and as having no exposure if they responded with ‘not at all’ for all the three sources of media) [35]. Scores are assigned to households based on the amount and types of consumer items they own, which can range from a television to a bicycle or automobile, as well as home features such as water supply, bathroom facilities, and flooring materials. Principal component analysis was used to calculate these scores. The national wealth quintiles are calculated by assigning a score to each typical (de jure) household member, rating each individual in the household population according to their score, and dividing the distribution into five equal groups, each having 20% of the population [34].

### Conceptual framework

We conceptualize our framework for this study based on the three-delay model of utilizing maternal healthcare services developed by Thaddeus and Maine [36] and then further elaborated by Gabrysch and Campbell to distinguish emergency care-seeking and preventive care-seeking [37]. The conceptual framework in this study captures the factors which determine the choice of place of delivery in terms of the first phase (delay in deciding to seek care), the second phase (delay in reaching adequate healthcare facility), and the third phase (delay in receiving quality care in a health facility).

**Fig. 1:**
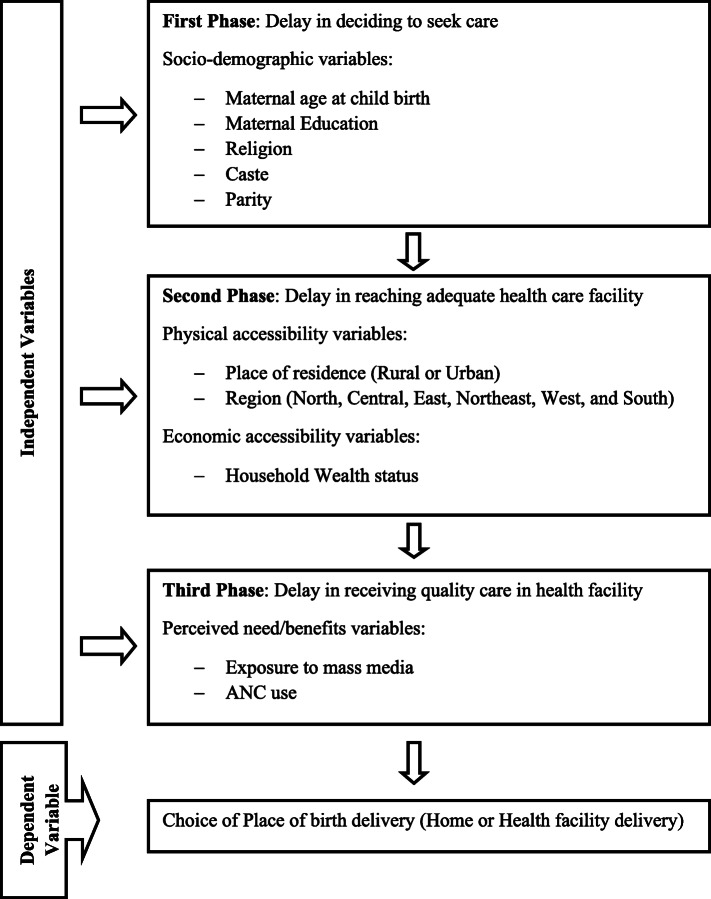
Conceptual framework for the determinants of the choice of place of delivery

According to the framework, the variables in the first phase include mother age at childbirth, maternal education, religion, caste, and parity. These factors are socio-cultural and demographic characteristics that influence the individual choice of access to and utilization of healthcare services. The second phase variables consist of physical accessibility and economic accessibility. The place of residence, the geographical region was framed as physical accessibility (i.e., availability of transport services, condition of the road, and distance to health facility), the household wealth status was framed as economic accessibility (i.e., affordability to bear the health care expenses). The variables in the third phase, such as exposure to mass media and ANC use was framed as perceived need/benefits. The conceptual framework of the study is shown in Fig. 1.

### Statistical analysis

The variables associated with home deliveries were assessed using bivariate and multivariate logistic regression analysis. In bivariate analysis, a chi-square test was used to examine the relationship between socio-demographic variables and house deliveries. In a multivariate analysis, factors that were statistically significant (*p* < 0.05) in bivariate analysis were included. The adjusted odds ratio was provided in the results, along with a 95% confidence range.

### Concentration index (CI)

The concentration index (CI) and the concentration curve (CC) were used to measure income-related disparity in home delivery, using the wealth score as the socio-economic indicator and the binary outcome as home delivery. Plotting the cumulative percentage of women giving birth at home versus the cumulative proportion of the population rated by the socio-economic indicator yields the concentration curve. The concentration index can be written as follows:

$$ \boldsymbol{C}=\frac{\mathbf{2}}{\boldsymbol{\mu}}\boldsymbol{\operatorname{cov}}\left({\boldsymbol{y}}_{\boldsymbol{i},}{\boldsymbol{R}}_{\boldsymbol{i}}\right) $$

Where C is the concentration index; *y*_*i*_ is the outcome variable index; ***R*** is the fractional rank of individual ***i*** in the distribution of socio-economic position; ***μ*** is the mean of the outcome variable of the sample, and ***cov*** denotes the covariance.

The concentration index takes a negative value if the curve is above the line of equality, suggesting a disproportionate concentration of inequality among the poor (pro-rich). If the curve falls below the line of equality, the concentration index is positive, suggesting that inequality is concentrated disproportionally among the wealthy (pro-poor). The concentration index is 0 when there is no socioeconomic disparity.

The concentration curve is estimated using following steps:

1. From the poorest wealth quintile to the richest wealth quintile, sort the wealth quintiles by the outcome variable (women delivering at home).
2. For each wealth quintile, calculate the number of women who give birth at home.
3. Determine what proportion of all women delivering at home is observed in each wealth quintile and what proportion of all women delivering at home is seen in each wealth quintile.
4. Determine the cumulative proportions of each variable.
5. Draw a graph with the X axis representing the proportion of total wealth (women delivering at home) and the Y axis representing the proportion of total women delivering at home.

### Decomposition of the concentration index

The concentration index was decomposed using Wagstaff decomposition technique. Wagstaff’s decomposition showed that the concentration index could be broken down into the contributions of each component to income disparities. Based on the linear regression relationship between the outcome variable *y*_*i*_, the intercept α, the relative contribution of *x*_*ki*_ and the residual error *ε*_*i*_

$$ {y}_i=\alpha +\sum {\beta}_k{x}_{ki}+{\varepsilon}_i $$

Where *ε*_*i*_ is an error term, given the relationship between *y*_*i*_ and *x*_*ki*_, the CI for y (C) can be rewritten as:

$$ C=\sum \left(\frac{\beta_k{\overline{x}}_k}{\mu}\right){C}_k+\frac{GC\varepsilon}{\mu }/\mu $$

Where *μ* is the mean of *y*_*i*_, ${\overline{x}}_k$, is the mean of *x*_*k*_, *β*_*k*_ is the coefficient from a linear regression of outcome variable, *C*_*k*_ is the concentration index for *x*_*k*_ (defined analogously to C, and GC_ɛ_is the generalized concentration index for the error term (*ε*_*i*_).

Here C is the outcome of two components: First, the determinants or ‘explained’ factors. The explained factors indicate that the proportion of inequalities in the outcome (home delivery) variable is explained by the selected explanatory factors, i.e., x_k_. Second, a residual or ‘unexplained’ factor $\left(\frac{GC\varepsilon}{\mu }/\mu \right)$, indicating the inequality in health variables that cannot be explained by selected explanatory factors across various socio-economic groups.

## Results

The socio-demographic characteristics of the study population in India is shown in Table 1. The number of women giving birth at home has decreased by 39.6%, from 58.5% in 2005–06 to 18.9% in 2015–16. In 2005–06, almost 8.2% of women were 25 or older when they gave birth, compared to 15.4% in 2015–16. Women with four or more children made up 27.8% of the population in 2005–06, but just 15.3% in 2015–16. Full antenatal care (ANC) was received by 19.5% of women in 2015–16, up from 11.2% in 2011–06. From 2005 to 06 (47.4%) to 2015–16, the percentage of women without a high school diploma fell significantly (27.6%).

**Table 1:** Socio-demographic profile of study population in India, NFHS-III & NFHS-IV

Background characteristics	2005–06		2015–16	
	Percentage	Sample size (n)	Percentage	Sample size (n)
**Women delivering babies at home**				
No	41.5	15,293	81.1	154,818
Yes	58.5	21,557	18.9	36,080
**Age at first birth**				
< 18 years	30.5	9132	13.0	23,627
18–24 years	61.3	22,829	71.6	135,243
25 or more years	8.2	4889	15.4	32,028
**Parity**				
First parity	26.4	10,394	33.6	61,807
Second parity	28.7	10,934	34.5	62,484
Third parity	17.2	6297	16.6	33,064
Four or more parity	27.8	9225	15.3	33,543
**Ante-natal care**				
No	21.0	7191	9.6	21,879
Partial	67.8	24,632	70.9	136,617
Full	11.2	5027	19.5	32,402
**Skilled birth attendant**				
No	50.2	16,182	16.6	37,685
Yes	49.8	20,668	83.4	153,112
**Mass media exposure**				
No exposure	30.9	8486	24.6	49,374
Exposure	69.1	28,364	75.4	141,524
**Educational status**				
No education	47.4	14,095	27.6	55,165
Primary	14.0	5251	13.5	26,712
Secondary	32.7	14,215	46.9	88,871
Higher	6.0	3289	12.0	20,150
**Caste**				
Scheduled Caste	20.0	6331	21.2	35,170
Scheduled Tribe	9.4	5733	10.3	37,889
Other Backward Class	40.0	11,858	43.6	74,060
Others	30.5	12,928	25.0	43,779
**Religion**				
Hindu	78.9	25,806	78.9	138,343
Muslim	16.4	5851	16.1	29,309
Others	4.8	5193	5.0	23,246
**Wealth index**				
Poor	45.8	12,622	44.5	90,521
Middle	19.6	7418	19.9	38,393
Rich	34.6	16,810	35.6	61,984
**Place of residence**				
Urban	26.8	14,527	29.7	47,833
Rural	73.2	22,323	70.3	143,065
**Region**				
North	12.8	6557	13.2	36,079
Central	28.0	7875	25.7	52,952
East	25.3	5847	25.4	39,243
Northeast	4.1	6965	3.9	28,825
West	12.9	4178	13.1	13,892
South	16.9	5428	18.7	19,907
**Total**	100.0	36,850	100.0	190,898

Table 2 represents bivariate and logistic regression analysis estimates for women delivering babies at home by their background characteristics in India. Women with age at first birth 25 years or more had a lower likelihood to deliver babies at home in comparison to women whose age at first birth was less than 18 years in 2005–06 and 2015–16 [AOR: 0.59; CI: 0.49–0.69] and 2015–16 [AOR: 0.76; CI: 0.76–0.82], respectively). Women with four or higher parity had higher odds of delivering babies at home compared to women with parity one in 2005–06 [AOR: 1.70; CI: 1.49–1.92] and in 2015–16 [AOR: 2.49; CI: 2.03–2.80]. Women with full ANC had a lower likelihood to deliver babies at home in comparison to women with no ANC in 2005–06 [AOR: 0.34; CI: 0.28–0.41] and in 2015–16 [AOR: 0.41; CI: 0.38–0.45]). In 2015–16 women with media exposure had a lower likelihood of delivering babies at home than women with no media exposure [AOR: 0.89; CI: 0.84–0.93]. Women from higher educational status had lower odds to deliver babies at home in comparison to women who had no education in 2005–06 [AOR: 0.33; CI: 0.26–0.42] and in 2015–16 [AOR: 0.44; CI: 0.39–0.49]). Women from the rich wealth index had a lower likelihood to deliver babies at home in comparison to women from the poor wealth quintile in 2005–06 [AOR: 0.41; CI: 0.34–0.48] and in 2015–16 [AOR: 0.64; CI: 0.61–0.76]). Women from rural areas had a higher likelihood to deliver babies at home in comparison to women from urban areas (2005–06 [AOR: 1.94; CI: 1.76–2.14] and 2015–16 [AOR: 1.12; CI: 1.06–1.18]). The regional differences in women delivering babies at home are pretty diverse, and significant change was visible in the last decade. In central India, the odds of delivering babies were high in 2005–06 [AOR: 1.15; CI: 1.01–1.32], whereas in 2015–16, the situation was opposite [AOR: 0.92; CI: 0.87–0.98] in reference to women from north India.

**Table 2:** Bivariate and logistic regression analysis estimates for women delivering babies at home by their background characteristics in India, NFHS-III & NFHS-IV

Background characteristics	2005–06		2015–16	
	Home delivery (%)	AOR (95% CI)	Home delivery (%)	AOR (95% CI)
**Age at first birth**	$		$	
< 18 years	74.9	Ref.	28.9	Ref.
18–24 years	54.8	0.86***(0.78–0.96)	18.7	0.95*(0.9–1.01)
25 or more years	25.5	0.59***(0.49–0.69)	11.4	0.76***(0.70–0.82)
**Parity**	$		$	
First parity	38.9	Ref.	9.0	Ref.
Second parity	49.1	1.49***(1.34–1.66)	15.4	1.63***(1.54–1.72)
Third parity	67.1	1.95***(1.72–2.21)	26.1	2.02***(1.9–2.15)
Four or more parity	81.6	1.70***(1.49–1.92)	40.5	2.49***(2.03–2.80)
**Ante-natal care**	$		$	
No	87.7	Ref.	43.2	Ref.
Partial	56.5	0.70***(0.62–0.8)	19.2	0.59***(0.55–0.62)
Full	16.1	0.34***(0.28–0.41)	5.6	0.41***(0.38–0.45)
**Mass media exposure**	$		$	
No exposure	81.9	Ref.	37.5	Ref.
Exposure	48.1	1.03(0.92–1.17)	12.8	0.89***(0.84–0.93)
**Educational status**	$		$	
No education	80.5	Ref.	36.6	
Primary	61.0	0.83***(0.73–0.94)	24.7	0.89***(0.84–0.94)
Secondary	35.1	0.70***(0.63–0.79)	10.8	0.68***(0.65–0.72)
Higher	7.0	0.33***(0.26–0.42)	3.1	0.44***(0.39–0.49)
**Caste**	$		$	
Scheduled Caste	65.2	Ref.	19.7	Ref.
Scheduled Tribe	80.7	1.34***(1.18–1.52)	30.0	1.08**(1.01–1.16)
Other Backward Class	59.7	1.71***(1.47–2.00)	17.8	1.40***(1.31–1.5)
Others	45.8	1.48***(1.33–1.65)	15.4	1.02(0.97–1.09)
**Religion**	$		$	
Hindu	58.0	Ref.	17.1	Ref.
Muslim	64.7	0.84***(0.73–0.96)	28.1	1.52***(1.43–1.61)
Others	45.5	1.03(0.89–1.19)	17.0	1.57***(1.46–1.7)
**Wealth index**	$		$	
Poor	81.5	Ref.	31.4	Ref.
Middle	58.9	0.75***(0.66–0.85)	13.4	0.82***(0.77–0.86)
Rich	27.8	0.41***(0.34–0.48)	6.2	0.64***(0.61–0.76)
**Place of residence**	$		$	
Urban	29.5	Ref.	9.7	Ref.
Rural	69.1	1.94***(1.76–2.14)	22.8	1.12***(1.06–1.18)
**Region**	$		$	
North	59.3	Ref.	14.6	Ref.
Central	77.1	1.15**(1.01–1.32)	26.6	0.92**(0.87–0.98)
East	69.6	0.73***(0.63–0.84)	27.8	1.37***(1.29–1.47)
Northeast	70.2	0.88*(0.76–1.01)	28.8	1.45***(1.35–1.57)
West	36.2	0.36***(0.3–0.42)	9.0	0.50***(0.45–0.55)
South	24.7	0.21***(0.17–0.24)	4.1	0.35***(0.31–0.39)
**Total**	**58.5**		**18.9**	

$*p* < 0.001 based on chi-square test of significance; ***p < 0.001, **p < 0.05, *p < 0.10; *AOR* Adjusted odds ratio; *CI* Confidence Interval; Ref: Reference category

**Fig. 2:**
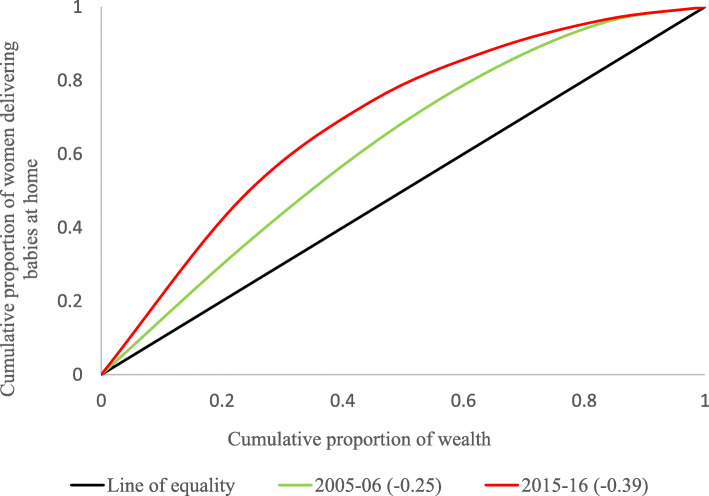
Concentration curve for women delivering babies at home in India, NFHS-III & NFHS-IV

Figure 2 provides the concentration curve for women delivering babies at home in India. It was found that the value of CI increased from − 0.25 to − 0.39 from 2005 to 06 to 2015–16; this depicts that the outcome variable (herein women delivering babies at home) got more concentrated among women from lower socio-economic status. This is a cause of concern as poorer women are at higher risk for delivering babies at home.

Table 3 provides decomposition analysis estimates for women delivering babies at home by their background characteristics in India. The first column is for coefficients from logistic regression analysis; the second and third columns are for elasticity and concentration index (CI), whereas the fourth column (absolute contribution) is the product of elasticity and CI. The fifth column (% contribution) is the proportion of absolute contribution multiplied by 100. The main aim of the decomposition analysis is to explain the percent contribution for socio-economic status (SES) related to inequality for women delivering babies at home. The wealth index explained 32.0 and 23.9% of SES-related inequality for women delivering babies at home in 2005–06 and 2015–16, respectively. Moreover, the educational status explained 18.6 and 18.9% of SES-related inequality, followed by residence (11.8 and 2.1%) and mass media exposure (2.7 and 12.9%) for women delivering babies at home 2005–06 and 2015–16 respectively. Additionally, parity explained5.4 and 13.4% of SES-related inequality for women delivering babies at home in 2005–06 and 2015–16, respectively. The region also explained 11.6 and 15.3% of SES-related inequality for women delivering babies at home in 2005–06 and 2015–16, respectively.

**Table 3:** Decomposition analysis estimates for women delivering babies at home by their background characteristics in India, NFHS-III & NFHS-IV

Background characteristics	2005–06					2015–16				
	Coefficient	Elasticity	CI	Absolute contribution	% contribution	Coefficient	Elasticity	CI	Absolute contribution	% contribution
**Age at first birth**										
< 18 years										
18–24 years	−0.220***	−0.02	0.07	0.00	1.1	−0.107***	−0.019	− 0.01	0.00	− 0.2
25 or more years	−0.728***	− 0.01	0.41	0.00	2.1	−0.336***	−0.005	0.24	0.00	1.8
**Parity**										
First parity										
Second parity	0.556***	0.03	0.15	0.00	−2.9	0.506***	0.019	0.11	0.00	−2.9
Third parity	0.842***	0.03	−0.02	0.00	0.3	0.764***	0.018	− 0.11	0.00	2.9
Four or more parity	0.859***	0.04	−0.26	− 0.01	8.0	0.947***	0.027	−0.35	− 0.01	13.4
**Ante-natal care**										
No										
Partial	−0.934***	−0.07	0.03	0.00	1.6	−0.924***	−0.109	− 0.04	0.00	−6.4
Full	−1.817***	−0.02	0.48	−0.01	8.3	−1.67***	− 0.036	0.31	−0.01	15.9
**Mass media exposure**										
No exposure										
Exposure	−0.217***	− 0.02	0.19	0.00	2.7	−0.265***	− 0.047	0.19	−0.01	12.9
**Educational status**										
No education										
Primary	− 0.311***	−0.01	− 0.02	0.00	− 0.1	− 0.168***	− 0.007	− 0.17	0.00	− 1.6
Secondary	− 0.682***	− 0.04	0.37	− 0.02	12.0	− 0.524***	− 0.043	0.18	− 0.01	11.3
Higher	−1.78***	−0.01	0.80	−0.01	6.7	−1.034***	−0.010	0.64	−0.01	9.2
**Caste**										
Scheduled Caste	0.316***	0.01	−0.15	0.00	1.1	0.067***	0.001	−0.13	0.00	0.2
Scheduled Tribe	0.723***	0.01	−0.41	0.00	3.5	0.397***	0.007	−0.36	0.00	3.4
Other Backward Class	0.34***	0.02	0.01	0.00	−0.1	−0.041**	0.000	0.03	0.00	0.0
Others										
**Religion**										
Hindu										
Muslim	0.154***	0.01	0.00	0.00	0.0	0.473***	0.013	0.03	0.00	−0.5
Others	−0.142***	0.00	0.21	0.00	0.1	0.45***	0.002	0.21	0.00	−0.7
**Wealth index**										
Poor										
Middle	−0.502***	− 0.01	0.16	0.00	1.6	−0.364***	−0.011	0.14	0.00	2.3
Rich	−1.132***	−0.06	0.68	−0.04	30.4	−0.738***	− 0.023	0.67	− 0.02	21.6
**Place of residence**										
Urban										
Rural	0.725***	0.10	−0.17	−0.02	11.8	0.197***	0.008	−0.18	0.00	2.1
**Region**										
North										
Central	0.137***	0.01	−0.14	0.00	1.4	0.281***	0.015	−0.12	0.00	2.6
East	−0.364***	0.00	−0.21	0.00	−0.4	0.244***	0.016	−0.33	− 0.01	7.6
Northeast	−0.154***	0.00	−0.07	0.00	0.1	0.547***	0.003	−0.17	0.00	0.6
West	−1.122***	−0.02	0.28	−0.01	3.7	−0.332***	− 0.004	0.23	0.00	1.1
South	−1.839***	−0.04	0.22	−0.01	6.8	−1.034***	−0.008	0.31	0.00	3.4
**Calculated CI**				−0.143	100.0				− 0.073	100.0
**Actual CI**				−0.247					−0.391	
**Residual**				−0.104					−0.318	

*CI* Concentration Index; ***p < 0.001, **p < 0.05, *p < 0.10

## Discussion

This article attempted to examine the risk factors associated with women delivering babies at home. Also, we tried to decompose the estimates for women delivering babies at home to examine the contribution of various factors contributing to baby deliveries at home. The results found improvements, over the decade, in the prevalence of women delivering a baby at home; it declined from 58.5% in 2005–06 to 18.9% in 2015–16. The decline in the prevalence of women delivering babies at home could be attributed to the improved maternal and child healthcare infrastructure over the two time periods [38–40]. Despite a decline in the prevalence of women delivering babies at home over the decade, the result noticed an increase in the concentration of women delivering babies at home towards the poor; it rose from − 0.25 in 2005–06 to − 0.39 in 2015–16. More poor women were delivering babies at home in 2015–16 than in 205–06.

Furthermore, this study noticed certain factors that were contributing to the risk of women delivering babies at home. Women with low age at first birth, with higher parity, without ante-natal care were more likely to deliver babies at home than their counterparts. Moreover, women who had mass media exposure, educated women, women from the richest wealth quintile household, and urban women were less likely to deliver babies at home than their counterparts. Mass-media exposure (12.9%), educational status (18.9%), and household wealth (23.9%) explained more than half (55.7%) of the socio-economic inequality in the prevalence of baby deliveries at home during 2015–16.

The results expectedly found a considerable decline in the prevalence of women delivering babies at home, a decline of around 40% from 58.5% in 2005–06 to 18.9% in 2015–16. This decline can be attributed to the improvements in maternal and child health care services that took place in the country after 2005–06 [31–41]. The age of the mother at their firstborn child is an important predictor of baby delivery at home. Results concluded that as the age of the mother at first birth increases, the odds of delivering the baby at home declines. In other words, as the age of a mother increases, there is a higher probability that she might choose institutional delivery over delivering her baby at home. Increasing maternal age may increase the perception of risk, thus reducing the chances of home delivery [42].

The results noticed that the higher the parity, the more likely the mothers would give birth at home. Previous studies also suggest that birth order or parity is an important driver of institutional delivery. With higher parity or birth order, chances of institutional delivery decrease among women, raising the odds of home delivery [43, 44]. The likely reason to choose home delivery by mothers with higher parity is that they perceive delivery as a normal process and develop the confidence to give birth at home [45]. It is plausible that after delivering the birth previously, subsequent deliveries are perceived to be of low risk, thus increasing the likelihood of delivering subsequent babies at home [42]. Women prefer to use skilled delivery care for their first delivery but then withdraw from utilizing skilled delivery services for subsequent births. This finding is interesting; however, it raises certain speculation for why women with higher parity do not prefer to use such services? It is because of previous unpleasant experiences with institutional delivery or factors related to the high cost associated with skilled care services or social practice [46]. Previous studies have noted that poor pregnancy experience during previous deliveries led to decreased maternal healthcare utilization in subsequent pregnancies [47–50]. However, further explorations are required to examine the reason for this finding.

Ante-natal care is another significant variable that affected the maternal choice of planning their delivery accordingly. Results from both periods noted that mothers who opted for ante-natal care were less likely to go for home delivery. Previous studies in various Indian settings are in line with this finding [42]. Studies conducted in other developing countries also concordance with this study’s finding [51, 52]. Women who opt for ANC are more likely to receive guidance from health professionals, prompting them to go for institutional delivery [52]. Furthermore, those who receive ANC from the beginning of their delivery care receive motivation to opt for SBA in institutional care during delivery [53, 54].

The study noticed the education status of the mother as a significant predictor of mothers delivering babies at home; mothers without any education were more likely to deliver their babies at home than educated mothers. This finding is consistent with studies from other developing countries [44, 45, 55, 56]. This finding is also concordant with studies from different settings in India [57]. Educated mothers are more likely to be aware of the hazards of home deliveries and therefore prefer institutional deliveries over home deliveries [57]. Furthermore, education promotes a better understanding of health messages and empowers women, enabling them to choose institutional delivery [43]. Education among women is positively associated with women’s autonomy [58], which is further linked to higher rates of institutional deliveries [59]. The association between women’s autonomy and institutional deliveries could be explained by the women’s relative position in the household relates to household decision-making [59]. When women are the decision-maker, they tend to choose better outcomes to utilize institutional healthcare services [60].

During both periods, the wealth index was noticed as an important factor affecting home delivery among mothers. Results concluded that richer women were less likely to deliver at home than their poor counterparts. Previous studies from India also revealed similar results for the association between household wealth and place of delivery [24, 61]. Women from poor households find it challenging to utilize SBA due to high out-of-pocket expenditures associated with institutional delivery and delivery at home [62, 63]. The poor utilization of SBA among the poor in India is a severe cause of concern as these services are supposed to be available to all free of cost at all government facilities [64]. The inequitable use of SBA between rich-poor raises questions regarding the availability, accessibility, quality, and cost incurred on utilizing SBA [46]. This study further noted that women from rural areas were more likely to deliver the baby at home than their urban counterparts. Previous studies in various Indian settings also agree with this finding [46]. The plausible factors can include lack of availability of skilled personnel, women’s reluctance or ignorance regarding using the services, or problems related to the poor quality of care in the rural area [65].

Socio-demographic factors such as household wealth, parity, mass-media exposure, and educational status of the mother contribute heavily in explaining the inequality to the prevalence of delivering babies at home in the decomposition analysis; these factors also appeared plausible predictors of home delivery in the logistic regression model. Household wealth and educational level of mothers were the two most prominent factors contributing to the inequality in the prevalence of deliveries at home during both the survey periods. Furthermore, results from the concentration curve revealed that most of the deliveries at home are concentrated among women in poor households, and the rich-poor gap has widened in a decade. Despite the introduction of the National Rural Health Mission (NRHM) and other incentive schemes such as the provision of free delivery care implemented in various states of India, many poor women are still delivering their babies at home.

The current study is sensitive to few limitations. First, the cross-sectional nature of the survey does not allow us to infer causality. Further, this research has not considered the factors of transportation or the distance to the delivery institution. A previous study noted that distance to health facilities could be a determinant of institutional delivery [66]. Despite the above limitation, this study made a reasonable attempt to examine the factors associated with home delivery among women in India.

## Conclusion

Given the encouraging evidence on the back of reduced prevalence of home delivery over the two-survey period, sustained policy efforts are need of the hour to achieve further reductions in the prevalence of home-based delivery. Based on our findings, we can conclude that there is a need to promote institutional deliveries, particularly for poor women, women with higher parity, uneducated women, and rural women. Despite various efforts promoted by NRHM, much work needs to be done in the rural parts of the country, as rural women were more likely to opt for home delivery than their counterparts. Also, further studies are required to comprehend women’s perception of not utilizing the SBA.

### Policy implications

Given the preponderance of home deliveries among the poorer section of society, every effort should ensure that a trained SBA attends poor women. Government should propitious her efforts in providing SBA to all pregnant women. ANC is the most concurring contact point for mothers to get relevant information about the risks and complications they may encounter during delivery [65], and therefore effort should be directed to provide full ANC as it would further improve the institutional deliveries. Multiple approaches are indispensable to spread awareness about the benefits of SBA utilization as women with higher parity preferred home deliveries. Targeted interventions are called for to bring improvements in rural areas. Also, providing required information related to the SBA to the uneducated women could bring a change. Involving ASHA to disseminate the information about the importance of SBA would be helpful.

## Data Availability

The data is publically available to everyone upon request. The data can be accessed from https://dhsprogram.com/methodology/survey/survey-display-355.cfm

## Abbreviations

ANC: Antenatal care
ASHA: Accredited Social Health Activist
CC: Concentration Curve
CI: Confidence Interval
JSY: Janani Suraksha Yojana
MoHFW: Ministry of Health and Family Welfare
NFHS: National Family Health Survey
NRHM: National Rural Health Mission
OBC: Other Backward Class
OR: Odds Ratio
PSUs: Primary Sampling Units
SBA: Skilled Attendant at Birth
SC: Scheduled Caste
ST: Scheduled Tribe
